# Land subsidence analysis using synthetic aperture radar data

**DOI:** 10.1016/j.heliyon.2023.e14690

**Published:** 2023-03-20

**Authors:** Rida Bokhari, Hong Shu, Aqil Tariq, Nadhir Al-Ansari, Rufat Guluzade, Ting Chen, Ahsan Jamil, Muhammad Aslam

**Affiliations:** a State Key Laboratory of Information Engineering in Surveying, Mapping and Remote Sensing (LIESMARS), Wuhan University, Wuhan, 430079, China; b Lulea University of Technology, Lulea, 971 87, Sweden; c School of Earth Science and Engineering, Majoring in Geodesy and Survey Engineering, Hohai University, Nanjing, China; d School of Geodesy and Geomatics, Wuhan University, 430072, Wuhan, China; e Department of Plant and Environmental Sciences, New Mexico State University, 3170S Espina Str., Las Cruces, NM, 88003-8001, USA; f School of Computing Engineering and Physical Sciences, University of West of Scotland, UK

**Keywords:** Land subsidence, Sentinel-1, SARPROZ, InSAR, PS-InSAR

## Abstract

Land subsidence is considered a threat to developing cities and is triggered by several natural (geological and seismic) and human (mining, groundwater withdrawal, oil and gas extraction, constructions) factors. This research has gathered datasets consisting of 80 Sentinel-1A ascending and descending SLC images from July 2017 to July 2019. This dataset, concerning InSAR and PS-InSAR, is processed with SARPROZ software to determine the land subsidence in Gwadar City, Balochistan, Pakistan. Later, the maps were created with ArcGIS 10.8. Due to InSAR’s limitations in measuring millimeter-scale surface deformation, Multi-Temporal InSAR techniques, like PS-InSAR, are introduced to provide better accuracy, consistency, and fewer errors of deformation analysis. This remote-based SAR technique is helpful in the Gwadar area; for researchers, city mobility is constrained and has become more restricted post-Covid-19. This technique requires multiple images acquired of the same place at different times for estimating surface deformation per year, along with surface uplifting and subsidence. The InSAR results showed maximum deformation in the Koh-i-Mehdi Mountain from 2017 to 2019. The PS-InSAR results showed subsidence up to −92 mm/year in ascending track and −66 mm/year in descending track in the area of Koh-i-Mehdi Mountain, and up to −48 mm/year in ascending track and −32 mm/year in descending track in the area of the deep seaport. From our experimental results, a high subsidence rate has been found in the newly evolving Gwadar City. This city is very beneficial to the country’s economic development because of its deep-sea port, developed by the China-Pakistan Economic Corridor (CPEC). The research is associated with a detailed analysis of Gwadar City, identifying the areas with significant subsidence, and enlisting the possible causes that are needed to be resolved before further developments. Our findings are helpful to urban development and disaster monitoring as the city is being promoted as the next significant deep seaport with the start of CPEC.

## 1 Introduction

Land subsidence is a geological hazard that can happen gradually and suddenly and is considered a threat to developing cities. It is triggered by several natural (geology and seismicity) and man-made (mining, ground-water withdrawal, oil and gas extraction, constructions, etc.) factors [1]. Land subsidence occurs naturally due to earthquakes, deformation due to volcanic activity, sediment compaction, land-sliding, groundwater withdrawal, underground mining, or oil and gas extractions [[2], [3], [4]]. It is a natural hazard that can progress gradually or suddenly depending on the conditions. Some earlier methods [[5], [6], [7], [8]] of measuring subsidence included a global positioning system (GPS), ground-based field observation data, repeated Geodetic Leveling Surveys, and ground and water sensors. However, these surveys are now considered costly and limited and present-day research relies on remote sending based Synthetic Aperture Radar (SAR) surveys for measuring subsidence [9,10]. Land subsidence was previously monitored through techniques such as point-based leveling, GPS, ground-based field observation data, repeated Geodetic Leveling Surveys (GLS), and ground and water sensors [9,11,12]. However, these techniques are costly and outdated, and their data is not easily available in Pakistan. Therefore, with the success of PS-InSAR techniques, land subsidence monitoring became more easily accessible [[13], [14], [15]].

InSAR is a powerful technique providing millimeter-level accuracy in the fields of deformation in the land. It helps monitor the surface changes continuously and is not affected by the weather [[16], [17], [18], [19]]. It has been widely used to detect surface changes due to subsidence and uplifting caused by both geological and anthropogenic factors [[20], [21], [22], [23]]. Although this technique has evolved and developed with time, apart from its capabilities, it was easily influenced by atmospheric effects [24]. In addition, it also faced problems in geometric, temporal, and phase decorrelation and phase unwrapping errors. These limitations demanded more advanced multi-temporal InSAR techniques [[25], [26], [27]].

These techniques can be applied in many fields and are most used to assess deformation due to seismic or volcanic activity, landslides, earthquakes, floods, etc. The process requires stacks of multiple images acquired at different times and works by identifying points on the ground, known as persistent scatters. Generally, building structures and rock outcrops provide more stable scatters [28]. Several algorithms of multi-temporal InSAR have been developed with time to create a continuous record of the displacement of SAR images in a specific time [29,30]. There are three basic categories based on the selection of master and slave selection [31]. The first technique PSI (or PS-InSAR), uses a single master image with multiple slaves to create interferograms. It uses coherent pixels that have stable phases and amplitude. The second technique, Small Baseline (SBAS), uses multiple master and slave images to create interferograms and small perpendicular baselines, to reduce the baseline decorrelation effect and use the distributed pixels [26]. The third category is the combination of both PS-InSAR and SBAS. The algorithms are distinguished based on distributed and persistent scatters [30]. The PS-InSAR approach used in this research is according to Ref. [32], which uses a single master and follows the amplitude dispersion criterion for pixel selection [33].

This research aims to provide the ongoing subsidence in Gwadar City and give rise to the factors affecting it. Land subsidence is a geological hazard that happens gradually, whose progress is unpredictable, and can cause sudden damage. Subsidence is dependent on subsurface lithology. The presence of fine-grained sand, silt, and clay are more susceptible to being compacted than coarse-grain sediments [34,35]. The primary cause of subsidence is generally considered groundwater withdrawal. The province of Balochistan is a dry region, consisting of deserts and dry hills, with less rainfall, and prolonged need droughts. Gwadar City is the highest populated city in Southern Balochistan. It is experiencing rapid urbanization and industrialization, which increases the water requirement drastically [36]. However, the reservoirs in Gwadar City are dry and salinized. There are minimal to no groundwater extractions. The citizens depend on desalination water plants, which directly filter the salt from the seawater to produce freshwater [37]. The salinization of soils, ground, and surface water is caused by sea-level intrusion due to rising sea levels. The sea-level rise is also responsible for depleting the coastlines of Pakistan, with up to −2.43 m/year regression in the East and 8.34 m/year regression in the South [38]. Tide gauges are located at Gwadar, Ormara, Karachi, and Creek sites.

Along with sea-level rise, other factors contributing to subsidence in Gwadar City are the increase in construction and urbanization, natural alluvium soil compaction, and geotectonic activity. The researchers [39,40] also provided the geohazard modeling of Gwadar City’s seismicity due to being located on the active Makran subduction zone. The city is not only subject to subsidence but is also vulnerable to tsunami risks. The damage analysis on the isthmus structure of Gwadar City showed results that the narrow strip of land extending on the sea (where most of the urbanization was found) is at the lowest altitude of only 0–10 feet [40] and is at risk of being completely inundated if an earthquake of 9 MW occurs.

InSAR processes have been the most integrated and functional tool in measuring the displacement of the earth’s surface on a millimeter scale. It is used worldwide and has widespread utilization in measuring displacement related to natural hazards, for example, earthquake, volcanic activity, tsunami, and landslides [38,41,42]. The process involves a high density of measuring points over remote, unapproachable, and risky areas. Unlike optical satellite imagery, the SAR sensor is independent of solar radiation and is not affected by weather conditions. There are several more advanced InSAR techniques, one of which is Multi-Temporal InSAR techniques like PS-InSAR. It provides better accuracy, consistency, and fewer errors in processing to overcome the limitations of conventional InSAR. In this technique, multiple images are acquired of the same place at different times to estimate the rate of surface velocity, along with surface uplifting and subsidence [41,43].

Therefore, the SAR analysis and modeling performed here in this research will provide information on the study area and help future researchers and developers for further assessment. The subsidence in Gwadar City is a recent event, increased from 2018 onwards, and is still progressing. The reported high sea-level rise and the chemically weathered and compressed alluvial sedimentary cover of Gwadar City have been the most alarming. These are two important factors contributing to the increasing subsidence in Gwadar City. The sea level of Gwadar City has the highest rise in the country and is constantly increasing during the research period. The research also reported tectonic land uplifting at Gwadar City’s coasts, up to 124 mm/yr. Gwadar City is situated at the Makran Accretionary prism associated with the active subduction zone between the Indian and Eurasian plates. The city is in Pakistan’s high seismic hazard zones and experiences high tides, causing seawater intrusion, coast erosion, and groundwater salinization. The main aim of this research is to analyze the progressive deformation in Gwadar City and provide its possible cause. Therefore, this research aims to provide: (a) SAR analysis using InSAR and PS-InSAR techniques to have both displacement and possible subsidence and uplifting analysis of the study area, (b) a study of the potential factors causing the displacement, (c) detailed analysis of the newly developed Gwadar City.

## 2 Materials and methods

### 2.1 Study area

Gwadar City is located in the South-West of Pakistan along the shores of Balochistan around 25°07′35″N to 62°19′21″E. It is one of the four cities of Gwadar District along with Pasni, Ormara, and Jiwani. The whole district has a population of only 300,000 people [44]. The Gwadar City structure is defined by a long narrow peninsula forming an isthmus extending up to 12 km, with 3–10 m in height from the mean sea level, extending Northward up to 50 m high [39]. The Gwadar Isthmus is formed between Pakistan and Gwadar Foreland, which hosts the gorge side. It includes a bay, which is up to 240 m wide, separating the Western bay called “Paddi Zirr,” which is up to 9.1 m deep, and the Eastern bay called Demi Zirr, which is the deep-water bay, providing the land to Gwadar port [45]. The Gwadar foreland consists of two of the highest peaks, known as Koh-i-Batel, which is up to 137 m high, spreading up to 11 km, and Koh-i-Mehdi, 340 m tall [3,10,40,42,46].

**Fig. 1:**
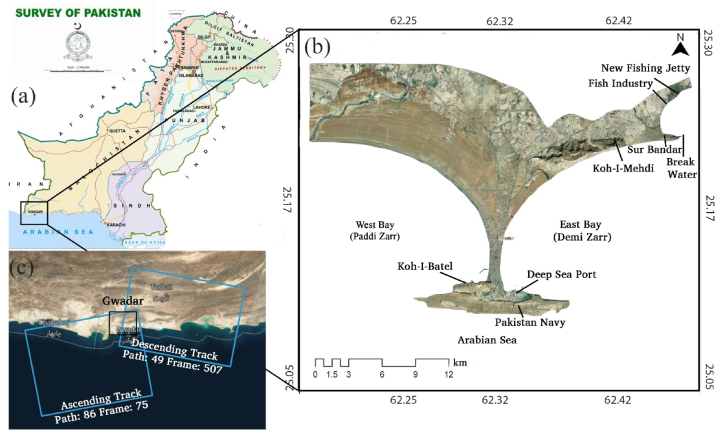
(a) Indicating the location of study area in Pakistan, (b) Gwadar district base map and (c) Satellite image courtesy of ESRI, Sentinel-1 data acquisition, descending and ascending tracks.

Gwadar City became the focus only recently; with the start of the China-Pakistan Economic Corridor (CPEC), this city is promoted as the next major deep seaport. The city is divided into two parts, East Bay and West Bay, and has two mountain peaks: Koh-e-Batil, 137 m tall, and Koh-e-Mehdi, 340 m tall [40]. Some reports suggest that Koh-i-Mehdi has two different height peaks 415 and 419 m. Fig. 1a-c displays the detailed study map of Gwadar City, indicating the essential features, including the location of the two highest mountain peaks of Gwadar, Koh-i-Mehdi and Koh-i-Batel, and the deep seaport with East and West Bay. It also displays the Gwadar District, featuring the four sub-districts and the location of the Makran subduction zone. Monitoring Gwadar’s land subsidence is essential because of its geology. The city is situated on the Makran Accretionary Prism, which consists of accumulated unconsolidated sediments, making it susceptible to deformation. Gwadar city is 51 km away from the central subduction zone, an active subduction zone between the plate boundaries of the Indian and Arabian plates [40]. The active tectonic zone highly influences the whole Gwadar district’s seismicity. This district consists of four cities: Pasni, Ormara, Jiwani, and Gwadar. The city’s coastal area is in a structure of Isthmus, extending onto the Arabian Sea. According to research Mahmood et al., [39], the narrow-shaped structure extending onto the sea is at risk of being entirely drowned if an earthquake of 9 MW hits the coast with a tsunami wave of more than 15 m. Some significant earthquakes have taken place in this district. The recent one was the 6.3 MW Pasni Earthquake on 7th February 2017, followed by a 5.2 MW aftershock on 8th February 2017. This location is Pakistan’s high seismic risk area, with an acceleration value of 0.12 g [47].

The future of Gwadar can be seen as the most advanced and the only adequately planned city in Pakistan. Several real estate and development scheme projects are already started, known as Green Palms, Canadian City Gwadar, and Gwadar Golf City, including Pakistan’s largest cricket stadium and Gwadar International Airport. Also, the Industrial zone of Gwadar will be fully functioning soon, along with the more developed fish harbor. It will also create employment opportunities for many.

### 2.2 Data collection, specifications, and acquisitions

The SAR data acquisition involves Electromagnetic Microwaves pulses (1 mm–1 m in wavelength, with 0.3 to 300-GHz frequency) transmitted from a space-borne SAR instrument, offering a high density of measuring points over the remote, unapproachable, and risky areas [[48], [49], [50], [51], [52], [53]]. InSAR technology has been the most integrated and functional tool in measuring the displacement of the earth’s surface on a millimeter scale. It is being used worldwide and has widespread utilization in measuring displacement related to naturally occurring events of as earthquakes, volcanic activity, tsunamis, and landslides [54]. The top right corner of Fig. 1 shows the descending and ascending paths of Sentinel-1A image acquisition over Gwadar City for this study.

This research uses Sentinel-1 Radar imagery of descending and ascending tracks to analyze the target area from two different positions by the satellite, with an incidence angle range of ∼23° vertically and ∼45° horizontally, from the East to West direction [48]. Using both of these tracks also helps better visualization of the area and proves land deformation through different directions [55,56]. The Sentinel-1 images are downloaded through the Alaskan Satellite Facility (ASF) using the European Space Agency (ESA). The Sentinel-1 images are available for public use in different types. Sentinel-1A, Single Look Complex (SLC) datasets are used for path 49 and frame 507 for descending track and path 86 and frame 75 for ascending track. It contains three Interferometric Wide Swaths (IW1, IW2 & IW3) and nine bursts [45,57]. This analysis is focused on IW3, and the coordinates of Gwadar city are adjusted. The complex burst images generated contain azimuth-time order into a single sub-swath image with demarcation and a black fill. The acquired Sentinel images are in VV + VH-polarization. The data consisted of a total of 80 images downloaded, 40 on descending track, and 40 on ascending track from July 2017 to July 2019. These images are utilized in SARPROZ for both the InSAR and PS-InSAR data processing.

The sea-level data of Gwadar City is obtained using two tide gauges from the world and sea level station monitoring facility. These instruments are located at Latitude 25.13 & Longitude 62.03 and Latitude 25.11 & Longitude 62.33, respectively. Both the devices follow UNESCO and consist of Radar sensors with a 1-min sampling rate. European Radar Observatory Sentinel-1 is a constellation of two satellites. Sentinel 1-A launched on 3rd April 2014, and Sentinel 1-B launched on 22nd April 2016. It was designed and developed by European Space Agency (ESA). The Sentinel-1 imageries can be acquired regardless of the weather and cloud cover because of their high-frequency acquisition and broad coverage. It allows the generation of high-quality interferograms for environmental hazard modeling, such as earthquakes, tsunamis, landslides, etc. It also allows risk and damage assessment and a fault rupture model [58]. The Sentinel-1A has a revisit time of 12 days. With the launch of Sentinel-1B, the time has been reduced to 6 days. These two satellites consist of four nominal operation modes, given in Table 1 below.

**Table 1:** Description of data sets were used in this study.

S. No	No of images	Duration	Specification	Polarization	Flight direction
01	40	2017-01-02 to 2018-07-08	S1A_IW_SLC	VV + VH	Descending
02	40	2017-03-17 to 2018-07-10	S1A_IW_SLC	VV + VH	Ascending

### 2.3 Data preparation

The first step of the process includes importing all the SLC data, both descending and ascending track data are imported and processed separately. The SARPROZ software selects the master image that meets the requirements of having a moderate climate during acquisition, and has a suitable position, for instance, in the middle of the star graph considering the perpendicular and temporal baseline. The area of interest was set according to the coordinates of Gwadar City along 25.2460 Latitudes and 62.2861 Longitude, and a total area of 10 km radius, and then the master and slave images were extracted and co-registered based on precision orbits and digital elevation models (DEM).

#### 2.3.1 Preliminary analysis and preliminary geocoding

The temporal standard deviation (the mean divided by the standard deviation matrix) is processed. The SRTM is downloaded automatically as the default DEM. The Amplitude-Phase Screen (APS), DEM errors, and orbit inaccuracies are estimated and removed to assess phase stability. The absolute amplitude values create disturbances in the processing, therefore during acquisition, it is considered that the pixels with similar amplitude will have a smaller amplitude [55,59,60].

In this step, the Reflectivity map is generated as the temporal average of all the images in the dataset. The averaging operation is applied, constraining the noise and intensifying the targets to have a stable reflectivity throughout the dataset. In SARPROZ, the main procedure of PS-InSAR is the PS-Points selection. The preliminary geocoding of the dataset is geocoded through the Ground Control Points (GCP) [50]. Here the height is automatically selected from the DEM, and the points are displayed on the reflectivity map generated. The next step is the co-registration refinement, where the co-registration is refined through the external DEM.

### 2.4 Preprocessing

SARPROZ is based on MATLAB and provides a Graphical User Interface (GUI) to make SAR processing easier. It is software designed to perform a wide range of SAR, InSAR, and Multi-Temporal InSAR processing. It has two versions, 1) the P-code version that runs on MATLAB and 2) the Compiled version that can run independently. It can be run on Unix, Windows, and Mac, and its P-codes version can run on Linux. It supports most satellite data, including Sentinel, MODIS, Envisat, Terra-SAR, etc. [7,13]. Therefore, SARPROZ being the commercial software, provides better results and accuracy. The SNAP software is designed to process Optical imageries and SAR imagery. In comparison, SARPROZ provided more exact results because it mainly focused on SAR processing. Furthermore, the processing steps in SARPROZ are more precise and detailed to minimize confusion. Nevertheless, each software performs SAR processing through different algorithms hence, providing different results.

#### 2.4.1 InSAR workflow

InSAR Processing involves selecting master and slave pairs in the single image interferogram processing. The absolute value of interferometric coherence helps with the correlation of nearby pixels, while the interferogram phase helps estimate the expected value of the interferometric phase [61]. The presence of vegetation and forests in the master and slave images causes depreciation of its correlation and overlooking its phase. Therefore, interferogram coherence is also used as a reliable indicator of phase, detection, and classification.

a) Goldstein Phase Filtering

The averaging operation applied on nearby interferogram pixels helps smooth out noisy phase variations and enhances the signal-to-noise ratio. This effect is also known as filtering for noise reduction, and the most used filter is Goldstein’s. Goldstein’s traditional low-pass filtering method smoothers the intensity of Fourier-transformed samples in overlapped interferogram patches and helps in clarifying the fringes [62].

b) Interferogram Flattening

The next step is flattening, it removes the flat terrain phase fringes, and is crucial for reconstructing DEM with high accuracy. The topographic phase components, which occur due to the variation of the range distance across images, are removed by DEM removal. It flattens the interferogram and only leaves the fringes that indicate the elevation changes.

c) Phase Unwrapping

Phase unwrapping is the process of integrating the fringes of an interferogram for decoding phase ambiguities in eq. (1):

(1) $$\emptyset_{i,k}^{UW}\left(s,l\right)=\emptyset_{i,k}\left(s,l\right)\pm 2n\pi$$

Here, “s,l” refers to the complex values of two images, “i,k” are the pixel notations, “$\emptyset^{UW}$” is the unwrapped phase, while “∅” is the wrapped phase. Thereby, the unwrapped phase is the same as the wrapped phase with the addition of an integer multiple of *2π*. High coherence interferograms are easier for phase unwrapping, while with low coherence, it becomes nearly impossible to unwrap [63]. In SAR imaging, the sensor’s signal “*g*” is detected in the complex consisting of phase and amplitude. It represents the different types of scatterers in the ground and topography in an interferogram. The signal is estimated by eq. (2):

(2) $$g=Ae^{-j\varphi }=Ae^{j\frac{4\pi }{\lambda }R}$$

The complex signal is represented by “g,” Amplitude is given by “A,” recorded phase by “j φ,” and the distance in the slant range direction, back and forth from the sensor to the scatterer is given by “*R*”.

The SAR image is formed when the electromagnetic signal is transmitted from the radar and reaches the scattered on the ground then returns to the radar (Two-way travel). The path of the electromagnetic signal to return from a scattered to where it was emitted is defined as the phase. It is considered as the sensor wavelength range from −1π to 1π and is used to recognize the position of the scattered in the slant range explained in eq. (3):

(3) $$ø=\frac{2\pi }{\lambda }2R=\frac{4\pi }{\lambda }R$$

The phase change “ø” between the transmitted and received signals is proportional to the two-way travel distance (back and forth from the sensor to the scatterer) “*2R*” of the radiation and is divided by the transmitted wavelength “λ” [64].

Scatterers from different distances from different slant ranges cause further delays between the transmitted and received signals. The equation will be negative when the signal represents phase delays, while the wavelength of the electromagnetic signal “λ” is measured as 5.6 cm for Sentinel -1A. Later the interpreted negative displacement values are derived from the phase values, determining movement away from the sensor, and positive values towards the sensor, in the slant range explained in eq. (4) [54,65].

(4) $$ø=-\frac{2\pi }{\lambda }2R=-\frac{4\pi }{\lambda }R$$

The negative phase change is due to the phase delays, “λ” represents the wavelength, “*2R*” is the two travel distances, back and forth from the sensor to the scattered. The two SAR images called the master (m) and the slave (s), are combined by multiplying the first image (master) with the complex conjugate of the second image (slave), to form an interferogram. The phase difference between the two SAR images is represented by the interferometric phase φ, which is by measured from using the complex conjugate of phase of the slave (s) image during multiplication [[64], [65], [66]]. The interferogram phase is written as eq. (5) below:

(5) $$\varphi =øm-øs=\frac{4\pi }{\lambda }\left(Rm-Rs\right)$$

Interferometric phase is calculated from the phase difference between the two SAR images, Master (m), and the Slave (s) and “R” is the distance back and forth from the sensor to the scatterer. The complex interferogram “Φ” is generated using the discrepancy between the two-phase signals using two complex SAR images by below eq. (6):

(6) $$\Phi =g_{m}g_{s}^{*}=A_{m}A_{s}e^{j\frac{4\pi }{\lambda }\Delta R}=A_{m}A_{s}e^{j\Delta \varphi }$$

Here, the Complex interferogram is “Φ”,“g” is the complex signal, the discrepancy between the two recorded phase signals “ jΔφ”, Amplitude is given by “*A*,” and “*R*” is the distance back and forth from the sensor to the scatterer.

The interferometric phase generated in an interferogram has variations in detected phase signals from one resolution cell to the other due to the differentiated noise contribution. Stable scatterers on the ground will give more reliable phases, unlike weak scatterers with dispersed phases. The phase noise occurs from the change in detecting amplitude value due to the atmospheric factor, temporal changes, and different look angles between the two SAR images.

d) Sparse LS Method

**Fig. 2:**
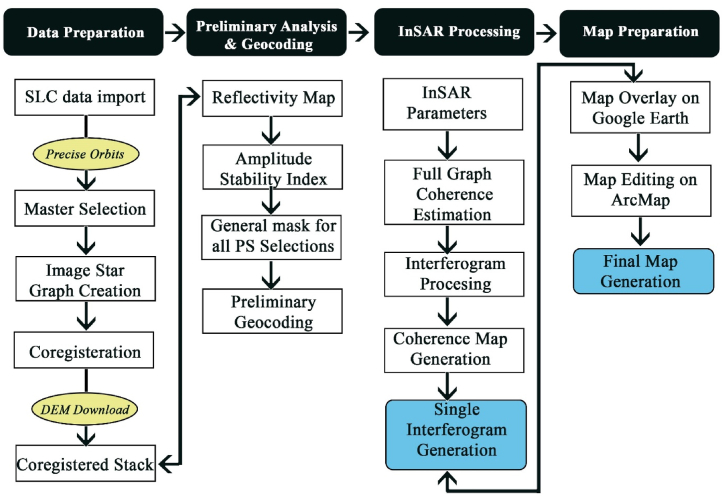
The InSAR workflow in SARPROZ.

The Sparse LS method is used in SARPROZ for the phase unwrapping method. It removes the unwrap fringes and uses sparse points and Least Squares to connect unconnected areas and solve the noise problem. The areas with coherence below the threshold are masked. The interferometric phase can be plotted either in height (m) or Displacement (mm). The simple SARPROZ workflow of InSAR is given in Fig. 2. A total of 8 images were used to create 4 master and slave pairs to generate interferograms with half-year intervals from July 2017 to July 2019.

#### 2.4.2 PS-InSAR processing and workflow

**Fig. 3:**
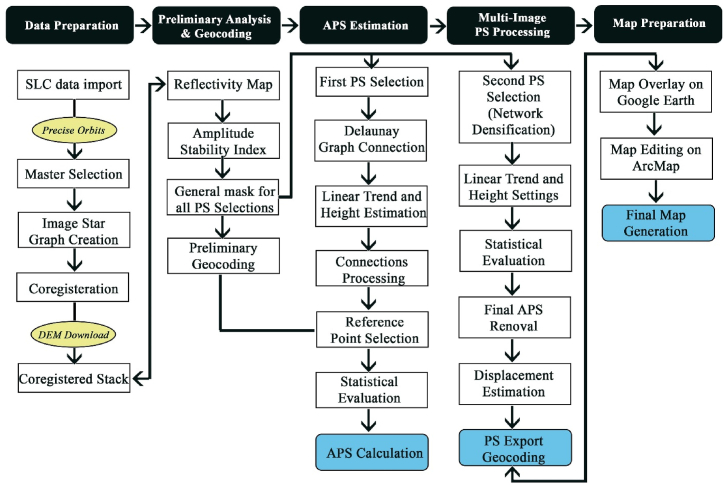
The PS-InSAR workflow in SARPROZ.

The Multi-Temporal InSAR technique of PS-InSAR uses a single master and requires more than 20 images at different times to estimate the surface velocity over the year [67]. It includes a selection of stable pixels (PS-Points) with stable amplitude and phase. They are dense in rock outcrops and urban areas and dispersed in forest and agricultural areas. Here, SARPROZ software is used for processing this technique. The workflow is described in detail below in Fig. 3. There are 80 images for this process, 40 on descending and 40 on ascending. The master image is selected by default through the software. It is the image that best fits the criteria of good weather conditions and is located as the center image of the star graph, with perpendicular and temporal baseline, as displayed in Fig. 3. The study area was manually selected, which focused on the city of Gwadar, from Latitude 25.7641 and Longitude 62.2512, within a 10 km radius. The main procedure in PS-InSAR involves estimating and removing APS and preventing orbit and DEM errors; it is described in detail below.

During acquisition, the SAR images are influenced by many factors, including atmospheric and signal delay due to the aerosol particles. Several spatial-temporal filters are applied for APS estimation to avoid these discrepancies in the resultant data. The first step of this procedure is selecting the First PS-point with a threshold of 0.8 for this research. According to Ferretti et al., 2007 [64], the recommended threshold is 0.75. It helps in obtaining correct APS estimations. Only a small number of points satisfies the strict parameter [60]. After the selection of PS Points, a reference network is established by connecting these points by Delaunay Triangulation. It is fundamental for estimating the atmospheric phase screen, later the second set of PS-Points are added in sparse points processing to further densify the network [68].

PS-Points connected according to the Delaunay Triangulation Principles are called a Delaunay Network. It is a constructed network with a low number of arcs resulting in lower displacement accuracy. If the arc is more extended than the maximum correlated atmospheric length, it will be removed, and the redundancy of the Delaunay network will be lowered. The Delaunay Triangulation is defined by a triangular network that follows the rule that the circumcircles of all the triangles in the network are empty without considering the lengths of the arcs [68,69].

From the result of this procedure, estimated linear displacement velocities and residual height are eliminated and APS is estimated from the remaining phases from graph inversion. It is also essential to select a reference point that contains the properties of residual histogram height value and the peak of the estimated velocity histogram at zero. This helps ensure that your reference point is on the ground and stable [70]. The quality of the estimated APS is assessed through the temporal coherence analysis of PS Points after the graph inversion and APS removal, through which most of the points in this process gave satisfactory results with a coherence value above 0.8.

### 2.5 Multi-image PS processing

In this step, the PS points are selected again, for which the threshold taken is 0.75, to attain densified points. In comparison, the rest of the parameters and reference points remain the same as for APS estimation. The selected pixels were geocoded and exported to be transformed into PS points. All obtained results are exported for final analysis and map generation.

### 2.6 Map preparations

The geocoded PS-Points are exported to Google Earth for final land subsidence Only the PS-Points with coherence above 0.8 were used for mapping. The data was later processed in ArcMap, where only the points containing negative values were mapped, and the points were classified using the natural breaks.

## 3 Results

This section discusses the InSAR and PS-InSAR analysis from 2017 to 2019, after every six months, consisting of 5 pairs for descending and 5 pairs for ascending tracks. PS-InSAR analysis from January 2017 to July 2019 resulted in a total of 80 images, 40 on descending track and 40 on ascending track. These analyses are processed in SARPROZ software and mapped using ArcMap 10.8.

### 3.1 Land deformation in Gwadar city

**Fig. 4:**
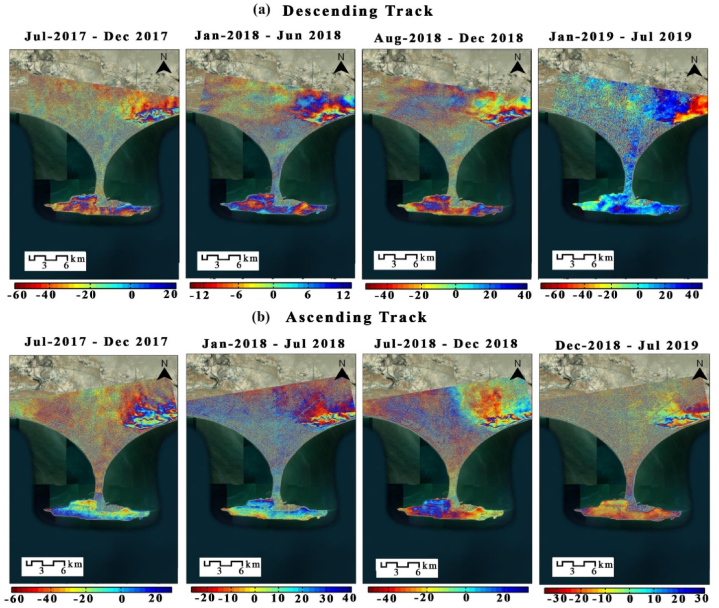
InSAR analysis of Gwadar city, (a) from the descending track and (b) ascending track. Processed in SARPROZ and converted into the map using ArcMap 10.8.

InSAR analysis of Gwadar City was done every six months from July 2017 to July 2019 from ascending and descending tracks. A total of eight images were used at a half-year interval. The results show the LOS phase of the enslaver and enslaved person pairs, with the deformation scale in millimeters. The area of Koh-i-Mehdi shows a change in fringes throughout the interferograms, indicating maximum displacement. Other areas on the map did not show significant deformation, resulting in ambiguity (Fig. 4a and b). The InSAR scale showed maximum deformation up to −60 mm in some intervals. The ascending track showed less deformation than descending one. The interferogram generated using SARPROZ is displayed in the results section. There are several components in processing InSAR for determining displacement through an interferogram. It begins with co-registration, ensuring that the same range and azimuth are contributed to each ground target in both the master and slave image. The software and the Digital Elevation Model (DEM) automatically download the orbit files using the default SRTM-3 Sec. It corrects all the remaining residual azimuth errors in the co-registration. This processing is done to the pairs of master and slave images to remove the temporal decorrelation, remove the appearance of burst bursts, and join all the bursts in a single image. The topographic phase removal is applied, which uses the DEM to remove all the possible phase impressions, and then the Goldstein Phase Filtering is applied. The displacement is calculated by analyzing the interferogram fringes using the color cycle. The interferogram fringe is a cycle of color range, with each cycle defined by the 2π cycle of phase change. There is a displacement even if the cycle contains only two colors [1].

The conventional way of performing an interferometric SAR is widely used worldwide. Due to its limitations in Phase decorrelation, processing Phase unwrapping error, and atmospheric artifacts hindrances, several new SAR techniques are introduced that provide better accuracy and fewer errors in processing.

### 3.2 Land subsidence at Koh-i-Mehdi

The results of land subsidence are obtained and further processed to determine the precise subsidence rates. The stability is determined by the stability threshold range, which is −3 mm/yr in descending and ascending tracks. All the points below the threshold are removed to avoid cluttering the data. The areas of significance in Gwadar City are processed separately for analyzing the cumulative displacement. The Line of Sight (LOS) motion is compared from both the tracks, where moving away from the sensor are negative points and towards the sensor are positive points. The points that display the same motion in both ascending and descending tracks are taken into observation to determine land uplifting and subsidence.

**Fig. 5:**
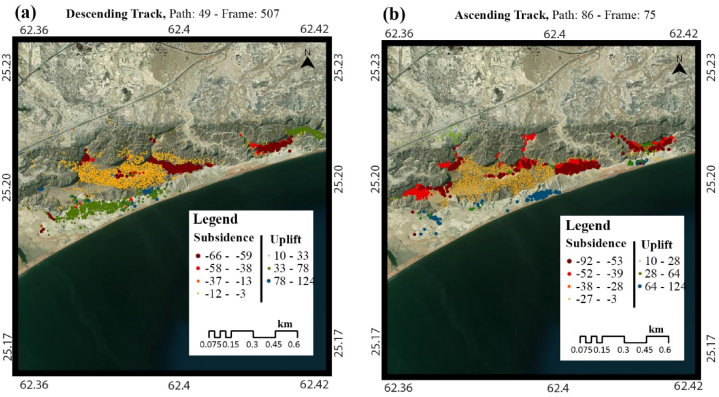
Land Subsidence Analysis of Koh-i-Mehdi area of Gwadar City in (a) descending track, (b) ascending track.

**Fig. 6:**
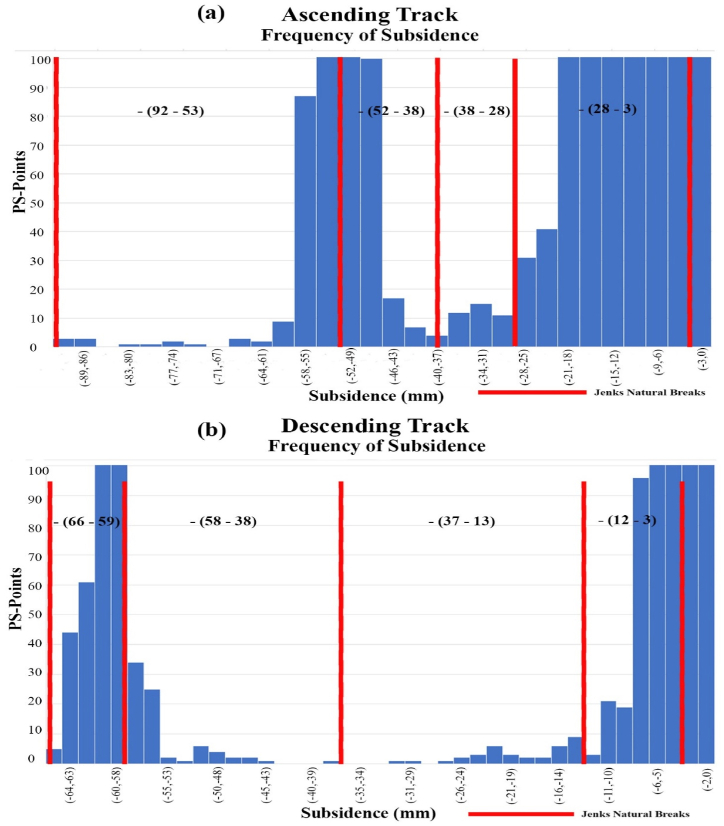
Frequency of Subsidence per PS-Points, with Jenks natural break, plotted in (a) ascending track, (b) descending track.

The ascending and descending track results from SARPROZ software were exported in .csv format and were processed for analysis in ArcMap 10.8. The subsidence point’s data is divided according to the Jenks natural break optimization, the method which selects the best arrangement for the data cluster. The frequency of subsidence points is displayed in Fig. 5(a,b). The points of specific areas are clipped by a polygon of the area and extracted to determine the maximum subsidence accurately. The maximum subsidence value observed around ascending track is up to −92 mm/year, while in descending track is −66 mm/year, as shown in Fig. 5, Fig. 6. The Koh-i-Mehdi area of Gwadar city located near the urban city and Surbandar consists of the Jiwani Formation containing limestone and sandstone, with a measured thickness of 100 ft. This formation is restricted to coastal areas and represents shoreline facies. Ormara Formation contains mainly soft and poorly consolidated sandy clay in the lower part, while the upper part is shell bearing. It has experienced the most subsidence and is increasing yearly, as shown in Fig. 5.

### 3.3 Land uplifting at Koh-i-Mehdi

**Fig. 7:**
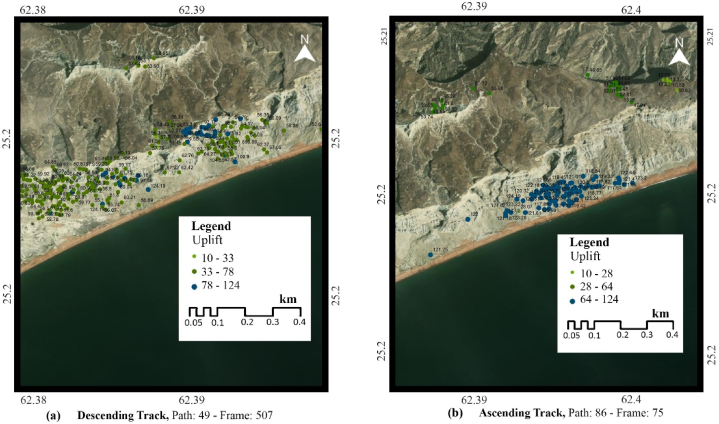
Land uplifting at the coast of Gwadar City in (a) descending track, (b) ascending track.

**Fig. 8:**
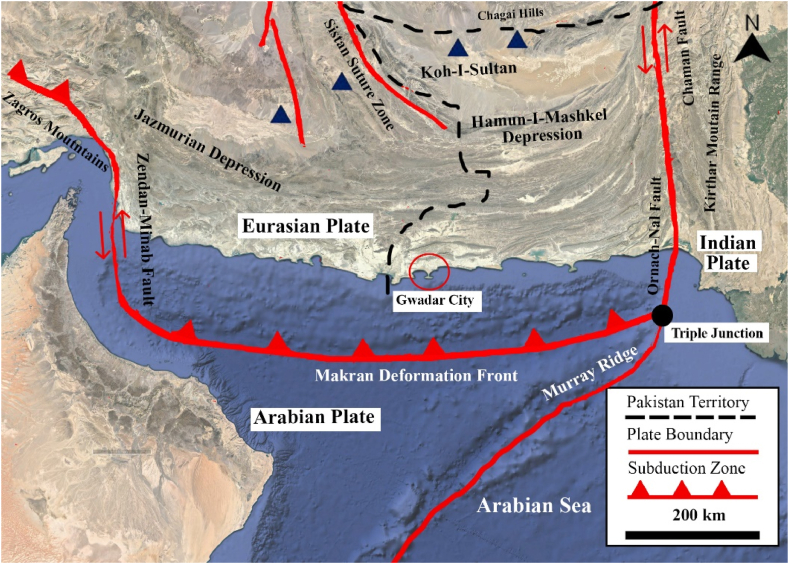
Makran Subduction Zone, along with the plate boundaries and location of Gwadar City.

The high uplifting zones in Koh-i-Mehdi are tectonic. Gwadar city is located on the Accretionary wedge active Makran subduction zone, the boundary between an Arabian and Eurasian plate with a 19.5 ± 2 mm/yr subduction rate. The oceanic Arabian plate is subsiding under the continental Eurasian plate causing uplifting in the coastal area of Gwadar city. According to PS-InSAR analysis in Fig. 7a, b, both ascending and descending tracks show uplifting up to 124 mm/yr. A more detailed look at the Makran subduction zone is given below in Fig. 8. It features the three plate boundaries of the Eurasian, Indian and Arabian plates, along with the vital fault zones and the location of Gwadar City on the map.

### 3.4 Yearly subsidence-rate

**Fig. 9:**
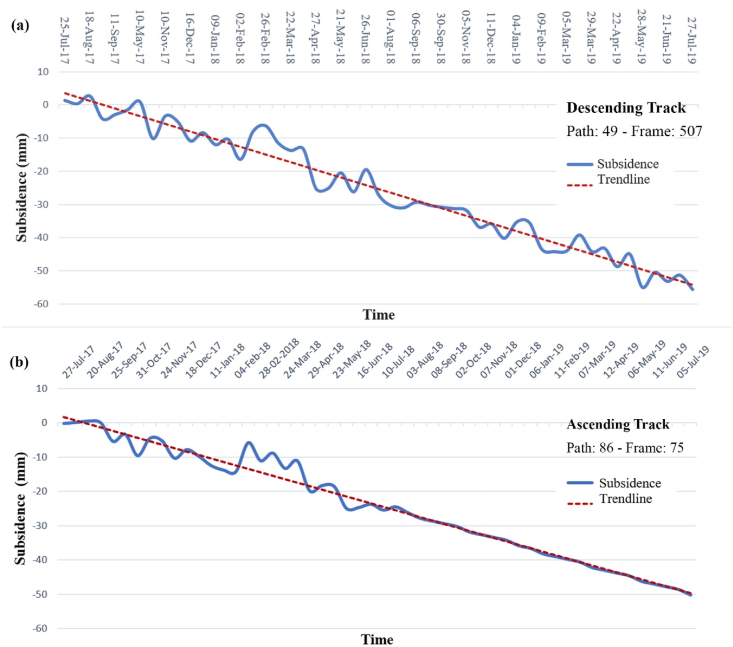
Yearly subsidence from the (a) descending and (b) ascending track in Gwadar City.

The PS-Points from the study area latitude 24.7641 and Longitude 62.2512, with a 10 km areal radius, are considered for determining the deformation trend. The subsidence results of 80 images are displayed, 40 in descending track from January 2017 to July 2018, and 40 in ascending track from March 2017 to July 2018. The subsidence trend is linear, the subsidence started from late-2017, and went steep downwards from late 2018, reaching up to 60 mm by early 2019. The subsidence in this region is a recent event and is still in the progressing stage (see Fig. 9a and b).

### 3.5 Gwadar sea-level

**Fig. 10:**
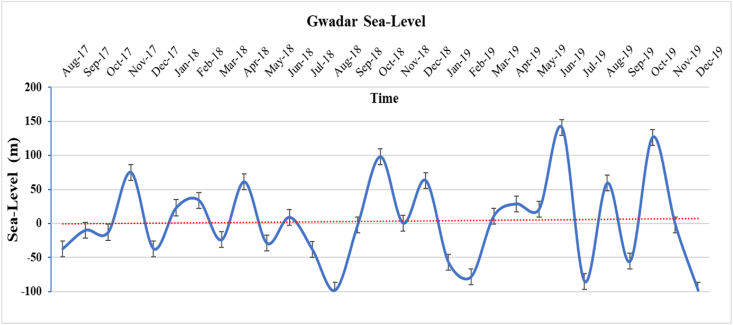
Gwadar sea-level rise the data.

The subsidence in Gwadar is directly related to the sea-water intrusion, a significant concern in coastal cities. This intrusion is associated with two factors: the area’s geology and the sea-level rise. As previously discussed, the water samples obtained from Gwadar contained high saline content, proving their sea-water origin. The geology of Gwadar also plays an important role in subsidence, the presence of high ${Na}^{+}/K^{+}$ the ratio in the ground sample was due to the presence of Chatti and Ormara clay formations, they have high potassium content, and are susceptible to chemical weathering. The Jiwani formation consists of limestone, which is composed of the mineral calcite and tends to dissolve by groundwater due to chemical weathering, resulting in caves and sinkholes. The other factor of sea-level rise in Gwadar is discussed below in Fig. 10. The data is obtained from the tide gauges near the city’s coast. Due to the presence of the coast, the sea-level rise rate in Gwadar is higher than in other cities. The data from July 2017 to Dec-2019 is displayed, which also covers the city’s subsidence period. According to the data, the noticeable increase in sea level begins after May 2019, with some peaks in mid-2018.

### 3.6 Subsidence at deep-sea port

**Fig. 11:**
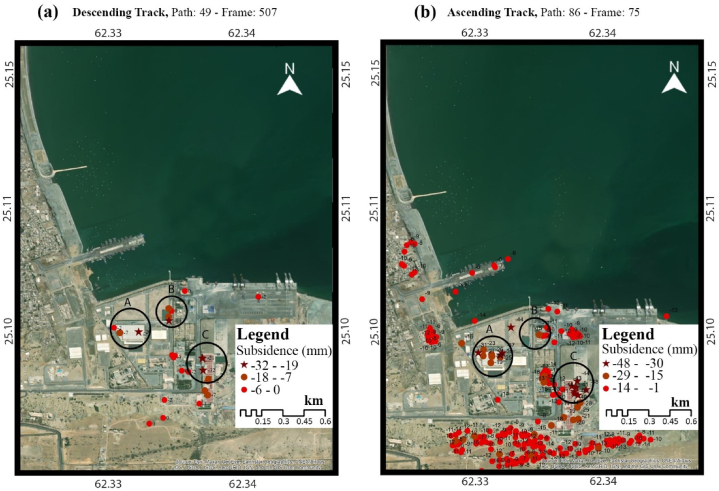
Subsidence analysis of Gwadar City deep seaport in (a) descending track, (b) ascending track.

Gwadar deep seaport is the center of all developments in Gwadar It is the main hub of CPEC, which is focused on the economy and development of both China and Pakistan. The giant desalination plant is also located in its vicinity, responsible for providing fresh water to the citizens. The subsidence data of the deep-sea port are displayed below in Fig. 11a. The highest subsidence on ascending track is −48 mm/year and on descending track is −32 mm/year. The of points on ascending track is higher than on the descending track. The Gwadar deep-sea port consists of the Jiwani formation, mainly near the shoreline, consisting of limestone and sandstone. The points A, B & C highlighted on the tracks are described in detail in the deep-sea port deep-seaport map in Fig. 11b. The map also highlights the important sites in the port.

## 4 Discussions

InSAR processes have been the most integrated and functional tool in measuring the deformation of the earth’s surface on a millimeter scale. It is used worldwide and has widespread utilization in measuring displacement related to natural hazards, for example, earthquake, volcanic activity, tsunami, and landslides. There are several more advanced multi-temporal InSAR techniques, one of which is PS-InSAR. It provides better accuracy, consistency, and fewer errors in processing to overcome the limitations of conventional InSAR. In this technique, multiple images are acquired of the same place at different times to estimate the velocity of uplifting and subsidence.

The data used in this research are discussed in detail in the results section, consisting of a total of 80 sentinel-1A images, 40 in descending track from January 2017 to July 2018 and 40 in ascending track from March 2017 to July 2018. These images are utilized in SARPROZ software for SAR processing. For this research, we used both ascending and descending tracks to provide two different-looking angles in the area for a better analysis, these tracks are processed separately, and then the results are compared. The results of SAR processing showed that the InSAR analysis helped detect the area that needed to be focused on for a more precise estimation of velocity. The area of Koh-i-Mehdi has a maximum change in fringes in all the interferograms of both descending and ascending, indicating deformation over time, with up to −60 mm deformation in LOS. The PS-InSAR analysis of the same area showed up to −66 mm/year subsidence on descending track and −92 mm/year on an ascending track. The map also displayed tectonic uplifting up to 124 mm/year in the coastal zone of Gwadar due to the Makran Subduction Zone. The area of the deep seaport was also analyzed, which showed −48 mm/year subsidence in ascending track and −32 mm/year subsidence in descending track. The rest of the area was below the stability threshold range and appeared stable in both tracks.

Despite being located in Pakistan’s high-hazard seismic zones, the city’s seismicity is relatively low compared to the neighboring sites. However, it does pose severe damage risk in the event of a high-magnitude earthquake. In comparison, urbanization is low in Koh-i-Mehdi where most of the subsidence has resulted. Therefore, it can be concluded that the subsidence has resulted from the alluvial sediments in the area since there is no ongoing ground water extraction. The Gwadar groundwater samples tested by Naseem et al., 2012 [36] reported high ${Na}^{+}/{Cl}^{-}$ and Mg/Ca ratios indicating seawater intrusion in the ground. The citizens rely on the desalinization plants to convert seawater into drinking water. According to the geology of the Gwadar city, the subsidence mostly showed in the Ormara formation consisting of poorly consolidated mudstone with sandstone, with a recorded thickness of 3000 ft present along with the sites of Koh-i-Mehdi. Also, there is the presence of the Jiwani formation consisting of limestone, conglomerates, and sandstones near the shoreline of Gwadar. Limestone is comprised of the mineral calcite and tends to dissolve in groundwater due to the process of chemical weathering, which results in caves and sinkholes. The coasts of Gwadar have also reported high tides and constant sea-level rise. These two are the main factors contributing to the increasing subsidence at Gwadar City, the compressibility of the sediments, and rising sea levels.

The main concern of Gwadar City is the lack of drinking water due to groundwater salinization. The city relies on the Ankara Dam for water supply. According to a report W.bank, 2013 [71], the water supply is less than 15% of the requirement, 1 million gallons every 2 days, compared to the required amount of gallons of 3.5 million/per day. The other factors were the seismicity of Gwadar City and the urbanization cover. Although these factors do not pose any severe risk, the study helped us understand that nearby seismicity impacts Gwadar City and the seaport. PS-InSAR is an essential technique for remote and underdeveloped areas yet to be studied thoroughly. This remote-based SAR approach was helpful in the Gwadar region because researcher access in the city is limited and has become even more so since COVID-19. Nevertheless, in the future, this research aims to be extended toward the current yearly subsidence rate. This analysis will also help locate the areas where reference GPS stations should be installed for future subsidence analysis.

The GPS data of the area for validation of the results was unavailable; several organizations were contacted to cooperate, but they have yet to volunteer. SAR processing, especially multi-temporal InSAR, requires high data processing to get accurate and large-area processing results. Most of the limitations in the processing came from the resources available, for example, low software and hardware performances. The analysis performed here is only till early 2019, covering only the peak changes in subsidence. A more updated subsidence analysis required computer laboratories, full access to software, and memory space.

This research focused on Gwadar City based on the new major projects and investments taking place, with the start of the China-Pakistan Economic Corridor (CPEC). This city is now being promoted as the next big deep seaport and is being rapidly urbanized and industrialized. Both Chinese and Pakistani businesses and investors are interested in Gwadar, seeing this city’s potential. However, the active subsidence in this city poses a major risk to future projects and questions the infrastructure of the city. These results from our research will help set some groundwork for more precise inspection, developments, and studies on Gwadar city, which will help predict and prevent future disasters. This will also help in improving the living conditions of the city.

## 5 Conclusion

This research helped to determine subsidence up to 92 mm/year on ascending track and 66 mm/year on descending track at Gwadar’s Koh-i-Mehdi Mountain, and up to 48 mm/year on ascending track and 32 mm/year on descending track at Gwadar’s deep seaport. The subsidence in Gwadar City is a recent event that has increased since 2018 and is still progressing. However, the area of Koh-i-Mehdi, where the subsidence is escalating, needs to be urbanized and projects less damage to the city. In contrast, the subsidence in the deep seaport is alarming. Among the factors affecting Gwadar, the reported high sea-level rise and the chemically weathered and compressed alluvial sedimentary cover has been the most critical. These two are the main factors contributing to the increasing subsidence at Gwadar. The city’s sea-level progression is also discussed, which is the highest sea-level rise in the country and is constantly creasing from 2017 to 2019 in the research period.

The research also has reported land uplifting at the coasts of Gwadar, up to 124 mm/year. This uplifting at the coast of Gwadar is tectonic. The city of Gwadar is located on the Makran accretionary prism, associated with the active Makran subduction zone, the boundary between the Arabian and Eurasian plates. Gwadar City is in the significant seismic hazard zone of Pakistan, with the risk of having severe damage in the event of an earthquake and has a risk of being wholly inundated in the event of a tsunami due to the coastal isthmus structure, high seismicity, and sea-level rise. Furthermore, the results may vary due to the need for more literature and data on the study area. However, this result will help set the groundwork for future, more definite results, and research.

## Author contribution statement

**Rida Bokhari, Hong Shu, and Aqil Tariq:** Conceived and designed the experiments; Performed the experiments; Analyzed and interpreted the data; Contributed reagents, materials, analysis tools or data; Wrote the paper. **Nadhir Al-Ansari, Rufat Guluzade, Ting Chen, Ahsan Jamil, and Muhammad Aslam:** Contributed reagents, materials, analysis tools or data; Wrote the paper.

## Funding statement

This research did not receive any specific grant from funding agencies in the public, commercial, or not-for-profit sectors.

## Data availability statement

Data will be made available on request.

## Declaration of interest’s statement

The authors declare no conflict of interest.
